# Effect of vitamin D supplementation on depressive symptoms and psychological wellbeing in healthy adult women: a double-blind randomised controlled clinical trial

**DOI:** 10.1017/jns.2018.14

**Published:** 2018-08-23

**Authors:** Maria A. Choukri, Tamlin S. Conner, Jill J. Haszard, Michelle J. Harper, Lisa A. Houghton

**Affiliations:** 1 Department of Psychology, University of Otago, Dunedin, New Zealand; 2 Department of Nursing, Midwifery, and Allied Health, Ara Institute of Canterbury, Christchurch, New Zealand; 3 Department of Human Nutrition, University of Otago, Dunedin, New Zealand

**Keywords:** Vitamin D, Depression, Anxiety, Flourishing, Mood, Randomised controlled trials, CES-D, Center for Epidemiologic Studies Depression Scale, HADS, Hospital Anxiety and Depression Scale

## Abstract

Epidemiological evidence has linked low vitamin D status to a range of mood disorders. However, studies examining whether vitamin D supplementation can improve mood-related outcomes in healthy populations are limited. We investigated whether vitamin D supplementation over winter is beneficial for improving mood-related outcomes in healthy women. A total of 152 healthy women (18–40 years) in Dunedin, New Zealand were randomly assigned to receive 50 000 IU (1·25 mg) of oral vitamin D_3_ or placebo once per month for 6 months. They completed the Center for Epidemiologic Studies Depression Scale, the anxiety subscale of the Hospital Anxiety and Depression Scale and the Flourishing Scale every month. Additionally, they reported their positive and negative mood each day for three consecutive days every 2 months. Participants provided a blood sample at the beginning and at the end of the study for 25-hydroxyvitamin D_3_ analysis. ANCOVA was used to compare the outcome measures between the groups, controlling for baseline. We found no evidence of lower depression (*P* = 0·339), lower anxiety (*P* = 0·862), higher flourishing (*P* = 0·453), higher positive moods (*P* = 0·518) or lower negative moods (*P* = 0·538) in the treatment group compared with the control group at follow-up. Mood outcomes over the study period were similar for the two groups. We found no evidence of any beneficial effect of monthly vitamin D_3_ supplementation on mood-related outcomes in healthy premenopausal women over the winter period, so recommendations for supplementations are not warranted in this population for mood-related outcomes.

Vitamin D is a fat-soluble vitamin synthesised in the skin in response to sun exposure, or, to a lesser degree, obtained from foods like mushrooms and fatty fish^(^^1^^,^^2^^)^. In the absence of supplementation or suitable food fortification, the concentration of circulating serum 25-hydroxyvitamin D decreases significantly during winter^(^^3^^)^. Suboptimal vitamin D status is increasingly common in Western countries^(^^4^^)^ as a modern lifestyle often reduces the time exposed to sunlight, decreasing opportunities to manufacture vitamin D in sufficient quantities^(^^5^^)^. In New Zealand, 2·9 % of adults are vitamin D deficient (25-hydroxyvitamin D ≤25·0 nmol/l), and another 27·1 % are below the recommended level of serum 25-hydroxyvitamin D (50·0 nmol/l)^(^^6^^)^. The prevalence is lower than some other Western countries such as Austria or Germany, and similar to the UK, France and the Netherlands^(^^7^^,^^8^^)^.

The role of vitamin D in physical health is well established. Vitamin D is involved in bone formation and mineralisation through its role in Ca homeostasis, and a low concentration of serum 25-hydroxyvitamin D/hypovitaminosis D is associated with increased bone turnover and bone loss^(^^9^^–^^11^^)^. In addition to the well-appreciated function in Ca homeostasis^(^^12^^)^, vitamin D has been implicated in the pathogenesis of several chronic diseases such as CVD, diabetes mellitus, and asthma, as well as all-cause mortality^(^^1^^,^^10^^,^^13^^)^. Vitamin D is now increasingly recognised for its neurosteroid activity and impact on brain serotonin^(^^1^^,^^14^^–^^16^^)^. Vitamin D regulates brain serotonin levels, and serotonin is a major neurotransmitter that underlies risk for depression^(17^^)^. Vitamin D status exhibits seasonal variation^(^^3^^,^^18^^,^^19^^)^, and this is similar to the seasonal variation of serotonin^(^^20^^)^. Additionally, there is evidence of increased markers of inflammation in anxiety, providing a biological connection between vitamin D and anxiety similar to that for depression^(^^21^^)^.

There is evidence that low vitamin D status may be associated with mood disorders, including major depressive disorder, seasonal affective disorder and premenstrual syndrome^(^^22^^,^^23^^)^. Several observational studies found a strong positive link between vitamin D status and depression status or subclinical depressive symptoms^(^^22^^,^^24^^–^^29^^)^. However, experimental evidence testing the effect of supplemental vitamin D on mood-related outcomes in a general population is limited.

Supplemental vitamin D is currently widely taken by the general population, especially during the winter period. While no statistics are available for vitamin D supplementation in New Zealand, US data show that about 35 % of men and 45 % of women take vitamin D supplements, an increase of 40 % during the last 20 years^(^^30^^)^. Supplementation with vitamin D has been shown to improve some aspects of physical health such as bone density^(^^11^^)^ and all-cause mortality^(^^31^^)^; however, there has been less research on the mood and mental health benefits of vitamin D supplementation in the general population^(^^26^^)^.

A few studies have also shown that vitamin D supplementation might improve depression and other mood outcomes in clinical population groups^(^^32^^–^^35^^)^. Yet the benefits of vitamin D supplementation to improve depression and mood in general (i.e. non-clinical) populations have not been fully investigated to date. One study has examined vitamin D supplementation (400 IU (10 μg), 800 IU (20 μg) or placebo) in a non-clinical sample of students aged between 18 and 43 years, and found that those randomly assigned to take a vitamin D supplement reported higher positive mood such as enthusiastic, excited, determined^(^^34^^)^; however, the study was limited by the relatively small sample (*n* 44) and very short duration (5 d).

Relatively little is known about the role of vitamin D and mood among young adults; recent analyses by our research group showed that lower vitamin D was associated with higher depression scores controlling for sex, age and ethnicity^(^^29^^)^. In this micro-longitudinal observational study, students aged 18–25 years reported on their wellbeing for 13 consecutive days and provided a blood sample for 25-hydroxyvitamin D analysis. The relationship between vitamin D and depression remained significant after adjusting for time spent outdoors and physical activity. Overall, these findings suggest that even among healthy young adults, vitamin D may be a significant risk factor for subclinical depressive symptoms. However, these findings were observational and it is not clear whether vitamin D is driving these mood differences *per se*.

This randomised double-blind placebo-controlled trial aimed to test the causal effects of vitamin D_3_ supplementation on depression in a large non-clinical sample of pre-menopausal women over a 6-month period. Additionally, the effect on anxiety, negative mood, positive mood and flourishing was investigated. No previous investigation has included such a wide range of measures of both negative and positive psychological outcomes in a single study. Whereas depression, anxiety and negative mood capture ill-being, positive mood and flourishing capture wellbeing. We investigated the effect of vitamin D supplementation in women because women report a higher frequency and intensity of depressive symptoms^(^^36^^,^^37^^)^ and are more likely to display seasonal variation in mood^(^^38^^)^. A measure of anxiety was also included as there is some evidence of a negative association between anxiety assessed using the anxiety subscale of the Depression, Anxiety, and Stress Scales (DASS-21) in pregnant women and serum 25-hydroxyvitamin D^(^^39^^)^. Anxiety may be correlated with depression^(^^40^^)^, and measuring both may explain if the previous results found in relation to depressive symptoms may be better explained by anxiety.

## Experimental methods

### Participants

This study was a double-blind, placebo-controlled, randomised clinical trial conducted from February 2013 to October 2013 in Dunedin, New Zealand (45° 52′0 S, similar to the latitude of Montreal, Canada; or Lyon, France in the northern hemisphere). Young adult women aged 18–40 years were recruited from February 2013 to April 2013 through posters, leaflets, Facebook posts and direct emails to staff of major employers in the city. Women who were not currently pregnant or breastfeeding, had access to the Internet, and were willing to provide a repeated blood sample were eligible for the study. Specific exclusion criteria included current/planned vitamin D supplementation (including as part of a multivitamin supplement); chronic liver and kidney disease; arteriosclerosis or cardiac function impairment; sarcoidosis and other possible granulomatous diseases; medication, including anticonvulsants, glucocorticoids and barbiturates that might affect vitamin D metabolism; and, overseas travel during the study period (February–October 2013). Based on a power analysis, we determined that a sample size of seventy-six persons per group would provide an 80 % chance of detecting a difference of five points in the average total Center for Epidemiologic Studies Depression Scale (CES-D) depression score between the groups at a two-sided significance (*P*) level of 0·05, with an assumed standard deviation of 9·67 (based on Crawford *et al*. 2011^(^^41^^)^), and a loss to follow-up of 20 %. A total of 152 participants were randomised into the study and randomly assigned to consume vitamin D supplements (*n* 76) or placebo supplements (*n* 76) for 6 months. The study was conducted according to the guidelines outlined by the Declaration of Helsinki. The University of Otago Human Ethics Committee (no. 13/031) approved the study, and informed written consent was obtained from all participants. The trial was registered with the Australian New Zealand Clinical Trials Registry (registration number: ACTRN12613000540718).

### Study design and procedure

The intervention was a 6-month randomised, double-blind, placebo-controlled, clinical trial. Participants were invited to complete an online screening survey. In total, 334 potential participants were screened for eligibility and 164 who fulfilled criteria were invited to make a booking for an initial onsite session using an online booking engine (http://scheduleonce.com). Potential participants who fulfilled the inclusion criteria and had none of the exclusion criteria (see above) were invited to an initial onsite session. Due to the gradual recruitment of participants, the study was run in three waves, with wave two starting 1 week after the first one, and wave three 2 weeks later. At baseline, sociodemographic, general health and measures of depression, anxiety and flourishing were collected using self-administered online questionnaires. For the next three consecutive days, participants completed online daily reports of positive and negative mood accessed via a nightly survey delivered to participants by email. Participants then visited the University of Otago Human Nutrition clinic for baseline measurements. Anthropometric data (height and weight) were collected using standardised techniques^(^^42^^)^. All measurements were taken by the same trained anthropometrist (M. A. C.) using calibrated equipment (Seca) with the participants wearing light clothing and no shoes. Participants also provided a baseline non-fasting blood sample and were randomised to receive either 6-monthly 50 000 IU (1·25 mg) vitamin D_3_ (cholecalciferol) supplement tablets or placebo.

**Fig. 1.**
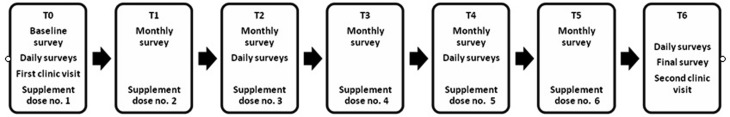
Timeline of the study.

Each month, participants completed the depression, anxiety and flourishing scales, which they accessed through an Internet survey link sent out in an invitation email. Immediately after completing the online surveys, they received a text reminder to take the monthly dose and confirm by return text that they had taken it. Every 2 months, participants rated their daily mood in additional ‘measurement bursts’ for three consecutive days via an online survey. At the endpoint, participants completed the final set of depression, anxiety, flourishing and daily mood surveys (Fig. 1), a retrospective questionnaire about time spent outdoors during the study period, and provided an endline blood sample for serum 25-hydroxyvitamin D analyses.

### Supplements

Supplements were provided in tablet form with instructions to be taken once per month. Participants received a text reminder to take their dose each month. The study supplements were manufactured in a single batch by Optimus Health from raw materials certified to contain no less than 100 000 IU/g (2·5 mg/g; equivalent to 100 % potency) supplied by a Food and Drugs Administration/Therapeutic Goods Administration-approved supplier of pharmaceutical-grade raw materials. The supplements were supplied as gelatine capsules, each containing microcrystalline cellulose as a filler and either 0 or 50 000 IU (1·25 mg) vitamin D_3_. The supplement and placebo pills were identical in size, colour and texture. Supplements were coded by a third party, the randomisation schedule was kept offsite, and all investigators remained blinded to the treatment until all statistical analyses were completed. The participants were provided with a sufficient supply of their randomly assigned tablets, and compliance was assessed by returned tablet count at the end of the study. There are no known adverse effects associated with taking a monthly 50 000 IU (1·25 mg) dose of vitamin D_3_ in healthy adult participants (equivalent to approximately 1667 IU (42 μg) of vitamin D per d). The level of vitamin D to be provided to participants is well below the tolerable upper intake level for adults set at 4000 IU (100 μg) per d by the Institute of Medicine^(^^43^^)^. Moreover, the standard tablet prescribed in New Zealand (subsidised by PHARMAC, the New Zealand government agency that decides which pharmaceuticals to publicly fund in New Zealand) is a 50 000 IU vitamin D_3_ monthly tablet.

### Measures

#### Demographics

Data on age, ethnicity and education were collected via a self-administered questionnaire during the initial onsite session.

#### Depressive symptoms

The presence of depressive symptoms was assessed using the CES-D^(^^44^^)^. This depression scale consists of twenty items that are answered with reference to the last week on a Likert scale from zero (‘rarely or none of the time’, i.e. less than 1 d) to three (‘most or all of the time’, i.e. 5 to 7 d). Items include statements such as ‘I thought my life had been a failure’ and ‘I had crying spells’. After reverse-scoring the four positively worded items, the scores for all items were summed to give a total score between 0 and 60 (Cronbach's α = 0·882). A cut-off score of ≥16 is recommended to indicate those who are at risk of depression. The CES-D was designed for use in community populations and focuses largely on the affective symptoms of depression. The CES-D is a validated instrument for the assessment of depressive symptoms and has been shown to be a reliable and valid tool for assessing the number, type and duration of depressive symptoms in a variety of populations, including women in the middle age between 36 and 67 years^(^^45^^)^.

#### Anxiety

Anxiety was assessed with the anxiety subscale of the Hospital Anxiety and Depression Scale (HADS)^(^^46^^)^. This scale was designed as a screening tool for anxiety and depression in general medical out-patients. It has good reliability^(^^47^^)^ and high convergent validity with other measures of anxiety^(^^48^^)^. Seven items answered with reference to the last week on a Likert scale from zero (not at all) to three assess anxiety, with the total score for the seven questions ranging from 0 to 21 (Cronbach's α = 0·651). Items included questions such as ‘Worrying thoughts go through my mind’. A score of eight or above on the subscale is recommended to indicate potential cases of depression and anxiety^(^^49^^)^.

#### Flourishing

Flourishing was assessed with the eight-item Flourishing Scale^(^^50^^)^ with items like ‘I lead a purposeful and meaningful life’ or ‘I am optimistic about my future’, that capture important aspects of a successful life including positive relationships, feelings of competence, and having meaning and purpose in life. Participants rated their general agreement with the statements on a scale from 1 (strongly disagree) to 7 (strongly agree). The total score ranges from 8 (strong disagreement with all items) to 56 (strong agreement with all items), with a higher score indicating greater flourishing (Cronbach's α = 0·941).

#### Positive and negative mood

At times 0, 2, 4 and 6 months, participants rated their daily positive and negative mood on a scale from 1 (not at all) to 5 (extremely) – with a nine-item positive mood/affect scale – happy, excited, cheerful, pleasant, calm, energetic, enthusiastic, content, relaxed, and a nine-item negative mood/affect scale – nervous, dejected, irritable, hostile, sad, angry, unhappy, anxious, tense across three consecutive days using an online daily survey. The nine positive and nine negative moods were combined into a composite measure of positive mood (nested α = 0·836) and negative mood (nested α = 0·785), respectively. The mood items are based on the affective circumplex that has been showed to reliably assess valence (positive *v.* negative) and activation level of mood (high, e.g. excited, medium, e.g. happy, and low, e.g. calm) and distinguish between them^(^^51^^,^^52^^)^. The daily positive and negative affect scores were averaged across each day, then across the 3-d measurement bursts each month for use as continuous outcome measures of positive and negative mood, respectively.

#### Anthropometry, time spent outdoors and skin colour

Participants’ height and weight were measured during the initial clinic visit, and BMI (kg/m^2^) was calculated. Participants reported retrospectively on time spent outdoors during the week and weekends. A score was allocated for time spent outdoors based on the work by Glanz *et al.*^(^^53^^)^. Skin colour was measured at the medial aspect of the upper arm using a Konica Minolta M-600d spectrophotometer and individual typology angle (ITA) was calculated for each participant based on previously developed formulae^(^^54^^)^. Skin colour was then classified into six physiologically relevant groups: ‘very light’, ‘light’, ‘intermediate’, ‘tan’, ‘brown’ and ‘dark’.

### Blood sampling and laboratory analysis

Non-fasting blood samples (4 ml) were drawn by venepuncture at baseline and follow-up (6 months later). The samples were collected into vacuum-evacuated serum collection tubes, and after 1 to 1·5 h, they were spun at 3000 rpm for 15 min at 4°C. Three aliquots of 0·5 ml serum per sample were transferred into standard micro test tubes (3810X; Eppendorf International) and frozen at −80°C until analysis without thawing or refreezing. Serum aliquots were batch analysed for serum 25-hydroxyvitamin D_2_ and serum 25-hydroxyvitamin D_3_ by the isotope-dilution liquid chromatography tandem MS method^(^^55^^)^, using an API 3200 instrument (Applied Biosystems) connected to a Dionex Ultimate 3000 HPLC system. The limit of quantification for the assay was <5 nmol/l. To assess accuracy and inter-assay variability, external quality-control serum material (UTAK Laboratories) containing low and medium 25-hydroxyvitamin D_3_ and 25-hydroxyvitamin D_2_ was analysed with every run. The 25-hydroxyvitamin D_3_ low control, verified value 29·9 nmol/l, mean was 27·2 (sd 1·5) nmol/l; CV 5·3 %, and the medium control, verified value 79·9 nmol/l, mean was 75·9 (sd 2·0) nmol/l; CV 2·6 %. For 25-hydroxyvitamin D_2_ the low control, verified value 26·6 nmol/l, mean was 23·8 (sd 1·7)  nmol/l; CV 7·3 % and the medium control, verified value 77·5 nmol/l, mean was75·9 (sd 2·8) nmol/l; CV 3·7 %. Internal quality-control pooled serum samples were also analysed; the inter-assay CV for serum 25-hydroxyvitamin D_3_ was 3·9 % at 44·8 nmol/l. The level of serum 25-hydroxyvitamin D_2_ in the internal controls was below the limit of quantification.

### Statistical analysis

The primary outcome of the trial was depressive symptoms measured with the total CES-D scores. Secondary outcomes included anxiety assessed with the anxiety subscale of the HADS, flourishing, as well as positive and negative mood. Data were analysed using an ANCOVA, comparing the continuous outcome measures (depression, anxiety, flourishing, positive and negative moods) at the end of the study between the groups, controlling for the baseline measures and covariates including age, ethnicity, BMI and recruitment wave. Means, standard deviations, regression coefficients, 95 % CI and *P* values were calculated. Although the study was not sufficiently powered to detect changes over time, we also ran a mixed-model linear regression analysis with random and fixed effects to assess whether there were any detectable differences in the trajectories of outcomes over time between treatment groups. This mixed model was run including time, plus the interaction term between time and treatment group. All data analyses were carried out using Stata version 12.0 (StataCorp).

## Results

### Participants and baseline measures

**Fig. 2.**
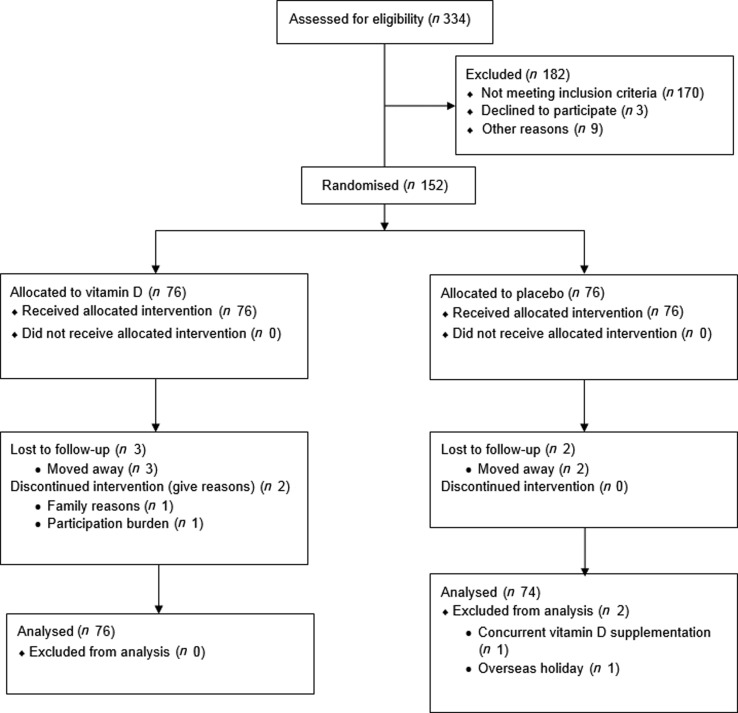
Flow of the study from screening to analysis.

The flow of study participants is shown in Fig. 2. A total of 334 recruits contacted the study team and were screened for eligibility. In all, 170 did not meet the inclusion criteria, three individuals declined to participate, and nine were excluded for other reasons (i.e. planning a holiday in a sunny destination). The remaining 152 eligible participants consented to participate. Seventy-six participants were randomly allocated to take vitamin D tablets, and seventy-six were allocated to take the placebo tablets. Five participants (3·3 %) were lost to follow-up (one in month 3, one in month 4, and two in month 5). Of these, three were in the vitamin D treatment group and two were in the placebo group. Intention-to-treat analysis was carried out including data from the above participants. One participant disclosed in the final survey that she had been taking a daily dose of vitamin D throughout the study, and the other participant disclosed that she travelled to a sunny destination for a holiday during the study period. Both participants were excluded from analysis.

The baseline characteristics of the randomised participants are shown in Table 1. The mean age was 24·2 (sd 6·0, range 18–40) years. The majority of participants were New Zealand European (80·7 %), and completing or having completed tertiary education (88·0 %). Most of the participants had very light (32·9 %) or light (53·3 %) skin colour. Of the participants, 23 % were overweight (25 > BMI < 30 kg/m^2^), 14·5 % were obese (BMI > 30 kg/m^2^), 2 % were underweight (BMI < 18·5 kg/m^2^) and 60·5 % were within the healthy BMI category (18·5 > BMI < 25 kg/m^2^). There were no significant differences in demographics, skin colour category or BMI observed between groups. There were also no significant differences between the groups at baseline in the outcome variables except flourishing, whereby the placebo group was lower than the vitamin D treatment group (41·6 *v.* 45·3; *P* = 0·021).

**Table 1.** Selected baseline characteristics of study participants (Mean values and standard deviations; numbers of participants and percentages)

	All (*n* 150)		Vitamin D (*n* 76)		Placebo (*n* 74)	
Variable	Mean	sd	Mean	sd	Mean	sd
Age (years)	24·2	6·0	24·4	6·4	23·9	5·6
Ethnicity*						
New Zealand European						
*n*	121		65		56	
%	80·7		85·5		75·7	
Māori/Pacific Islander						
*n*	7		4		3	
%	4·7		5·3		4·1	
Asian						
*n*	18		7		11	
%	12·0		9·2		14·9	
Other†						
*n*	4		0		4	
%	2·7		0·0		5·4	
Education						
Completed some high school or apprenticeship						
*n*	18		9		9	
%	12·0		11·8		12·1	
Completed or completing tertiary study						
*n*	132		67		65	
%	88·0		88·2		87·8	
Weight (kg)	68·4	14·1	68·7	12·8	68·1	15·4
BMI (kg/m^2^)	24·9	4·7	25·1	4·5	24·8	4·9
Body fat (%)	30·8	8·2	31·4	7·7	30·1	8·7
Constitutive skin colour (ITA)	51·0	10·5	51·3	12·5	50·7	8·3
Mean time spent outside score – weekdays‡	3·2	1·1	3·2	1·2	3·2	1·1
Mean time spent outside score – weekends‡	4·1	1·6	4·2	1·5	4·0	1·6
CES-D score	12·9	8·3	12·8	8·8	13·0	7·9
Anxiety score	13·1	3·0	13·1	2·9	13·0	3·0
Flourishing score	43·5	8·6	41·6	11·6	45·3	7·1
Positive mood	3·0	0·7	3·1	0·7	3·0	0·7
Negative mood	1·6	0·5	1·6	0·4	1·6	0·5
Serum 25-hydroxyvitamin D_2_ (nmol/l)	0·15	1·09	0·10	0·89	0·20	1·26
Serum 25-hydroxyvitamin D_3_ (nmol/l)	75·7	26·2	77·6	26·0	73·8	26·3
Total serum 25-hydroxyvitamin D (nmol/l)§	75·9	26·1	77·7	26·1	74·0	26·1
Waveǁ						
Wave 1						
*n*	63		33		30	
%	42·0		43·4		40·5	
Wave 2						
*n*	57		22		35	
%	38·0		29·0		47·3	
Wave 3						
*n*	30		21		9	
%	20·0		27·6		12·2	

CES-D, Center for Epidemiologic Studies Depression Scale; ITA, individual typology angle.

* Ethnicity categories based on top level categories defined by Statistics New Zealand: New Zealand European, Māori/Pacific Islander, Asian, other.

† Includes Latin American, Canadian First Nations and Ethiopian.

‡ Data collected retrospectively at the end of study to reflect the typical time spent outside during the study period.

§ Total serum 25-hydroxyvitamin D (serum 25-hydroxyvitamin D_2_ + serum 25-hydroxyvitamin D_3_).

ǁ The specific week-group in which the participant started the study.

Mean concentration of total serum 25-hydroxyvitamin D in all participants at baseline was 76·1 (sd 26·7; range 14·5–151·0; median 72·4) nmol/l. Of the participants, twenty-five (16·4 %) had serum 25-hydroxyvitamin D concentrations below 50 nmol/l, the cut-off used for sufficiency by the Institute of Medicine^(^^43^^)^. There was no significant difference between the groups in baseline total serum 25-hydroxyvitamin D (77·7 (sd 26·1) *v.* 74·0 (sd 26·6) nmol/l; *P* = 0·534). At the end of the study, the mean total serum 25-hydroxyvitamin D for the placebo group (49·2 (sd 28·1) nmol/l) showed the expected seasonal pattern in terms of a decline in absolute level and change over the seasonal periods (summer to winter) while serum 25-hydroxyvitamin D concentrations for the vitamin D group was statistically significantly higher (84·5 (sd 22·7) nmol/l; *P* < 0·001).

The mean baseline depression score for the sample was 12·9 (sd 8·3), which is similar to previously published normative data for the CES-D scale in this age range^(^^41^^)^. Conversely, the mean of the anxiety subscale of the HADS at baseline was 13·1 (sd 3·0), which is substantively higher than the normative mean of 6·96 (sd 4·19) for women in a non-clinical population established by Crawford *et al.*^(^^56^^)^. The mean baseline score of 43·5 across all participants on the Flourishing Scale is similar to 44·97 (sd = 6·56) found in the original validation study^(^^50^^)^.

### Adherence to intervention and missing data

Out of the ninety-eight participants who returned their supplement containers at the end of the study (65·3 % of the 150 participants included in the analysis, forty-seven vitamin D group, fifty-one placebo group), only three participants had one remaining supplement each (3 %, two vitamin D, one placebo).

There were no missing data at baseline for the demographics, serum 25-hydroxyvitamin D, depression, anxiety, or flourishing, with the exception of one participant missing one question on the twenty-item CES-D. This was dealt with through mean imputation of individual means. The mean number of daily mood surveys completed was 11·6 out of twelve surveys across all participants (96·7 (sd 1·1; range = 9–12) % compliance), with 128 participants having completed all twelve mood surveys during the study period (100 % compliance). At the first monthly follow-up, three participants missed a question each on the CES-D, one participant missed one question on the Flourishing Scale, and one participant missed all the anxiety questions. Individual means were imputed for the missing data based on the remaining items within the same scale for the current month; or the mean from the previous month was carried forward.

### Intervention effects

**Fig. 3.**
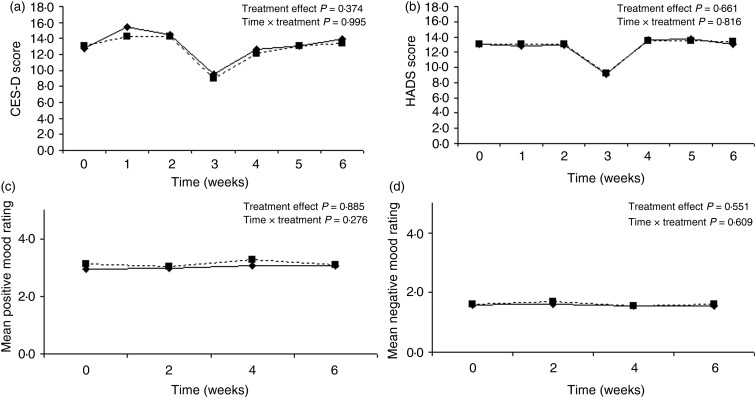
Graph of depressive symptoms (Center for Epidemiological Studies Depression Scale; CES-D) (a), anxiety (Hospital Anxiety and Depression Scale; HADS) (b), positive mood (mood circumplex) (c) and negative mood (mood circumplex) (d) plotted over time for the vitamin D group (–––) and placebo group (----) showing almost identical trajectories in outcomes over the study period.

The comparison of all outcome measures between the vitamin D and placebo groups at baseline and final measurement is shown in Table 2. There were no statistically significant differences between the vitamin D and placebo groups in any of the outcome measures – depression, anxiety, flourishing, or positive and negative mood, controlling for the baseline measures and the covariates. In addition, there were no differences in the trajectories over time between the treatment *v.* control group (i.e. no treatment or treatment × time interaction effects for any of the outcomes) (Fig. 3). Despite being underpowered to detect a statistically significant difference across the time points, the trajectories illustrate that a clinically significant difference is unlikely. Inclusion of multiple imputation estimates for the five missing values did not alter the results (imputed using twenty multiple imputation estimates for the regression and the results pooled). Similarly, when wave was excluded in the regression models for the primary analyses, the results remained the same.

**Table 2.** Comparison of outcome between the groups at baseline and final assessment, adjusted for wave (Mean values and standard deviations; effect sizes and 95 % confidence intervals)

	Vitamin D				Placebo						
	Baseline		Final		Baseline		Final				
	Mean	sd	Mean	sd	Mean	sd	Mean	sd	Effect size*	95 % CI	*P*
CES-D	13·1	7·9	13·6	10·1	12·7	8·9	13·9	9·9	−1·41	−4·33, 1·50	0·339
HADS anxiety subscale	13·0	3·0	13·4	3·0	13·2	2·9	13·2	2·9	0·16	−0·62, 0·95	0·862
Flourishing Scale	45·3	7·1	44·7	10·1	41·9	11·5	44·1	9·4	−1·08	−3·91, 1·75	0·453
Positive mood†	3·1	0·7	3·1	0·8	2·9	0·7	3·1	0·8	−0·07	−0·28, 0·14	0·518
Negative mood‡	1·6	0·4	1·6	0·6	1·6	0·5	1·5	0·5	0·05	−0·11, 0·21	0·538

CES-D, Center for Epidemiologic Studies Depression Scale; HADS, Hospital Anxiety and Depression Scale.

* Effect size estimate compares the difference in the outcome measure between baseline and endline between the two groups. Effect size <2 = small, 5 = medium, 8 = large.

† Mean composite of nine positive mood adjectives reported daily for three consecutive days.

‡ Mean composite of nine negative mood adjectives reported daily for three consecutive days.

## Discussion

In spite of previous studies showing an observational relationship between vitamin D and depression, the present study did not provide evidence for the benefit of single monthly dose of vitamin D_3_ supplementation over autumn and winter on depression and other mood outcomes in healthy pre-menopausal women. These null effects occurred despite several strengths of the present study design. Compared with previous studies, a wider variety of outcome measures at both the lower end of the wellbeing continuum (depression, anxiety) and the upper end of the continuum (flourishing) were included. Also, real-time positive and negative mood was measured, yet no benefit of vitamin D_3_ supplementation over winter was found in this population.

There are several plausible biological reasons supporting the potential role of vitamin D in the improvement of depression and mood. First, vitamin D receptors are present in brain regions involved in reward. Second, the key enzyme for the conversion of 25-hydroxyvitamin D to the active form, 1α-hydroxylase, is also present in the brain^(^^14^^)^. Indeed, the results of correlational studies suggested that vitamin D is a good predictor of depressive symptoms and mood^(^^29^^,^^57^^,^^58^^)^. Despite these biologically plausible pathways, results of this randomised controlled trial showed no evidence that monthly vitamin D supplementation improved psychological outcomes of women over winter.

Results of intervention trials for vitamin D have been inconsistent so far. However, the results of trials are difficult to compare because each differ considerably in terms of form of vitamin D used, dosage, frequency of administration, duration of participant follow-up, use of placebo controls, outcome measurements and sample size and population characteristics. While the monthly supplement regimen used in the present study was efficacious in maintaining vitamin D status over the winter, the pharmacokinetics of monthly *v.* daily supplementation are uniquely different. A single high dose leads to higher mean serum 25-hydroxyvitamin D, while a lower dose administered daily leads to a longer period when the level is maintained^(^^59^^)^. Previous trials investigating the effect of vitamin D have used various dosage regimens. For example, Bertone-Johnson *et al.* used a daily dose of 400 IU (10 μg) in combination with Ca *v.* placebo, but saw no effect on depressive symptoms^(^^32^^)^. The authors of another study using vitamin D in combination with oestrogen also saw no effect on depressive symptomatology^(^^60^^)^. Högberg *et al.* provided participants with a higher vitamin D dose – 4000 IU (100 μg) daily for the first month and 2000 IU (50 μg) daily for the next 2 months^(^^61^^)^. In that study, the authors did find a decrease in depressive symptoms. Nevertheless, the study population consisted of clinically depressed participants with a concurrent low serum 25-hydroxyvitamin D at baseline, so it is difficult to disentangle whether the effect was due to the higher dose used, or to the specific characteristics of the population. In our sample, the majority of participants had sufficient vitamin D at the start of the study. Contrary to Högberg's results, Frandsen *et al.*^(^^62^^)^ found no effect of supplementation in a sample with a slightly elevated score of seasonal affective disorder. The authors used a dose of 2800 IU (70 μg) per d. Nevertheless, their sample consisted of only forty-three participants and they experienced a high level of attrition, and, as a result, the study may have been underpowered to detect a difference. Dean *et al.* provided participants with a daily dose of 5000 IU (125 μg), yet, no difference was seen in depressive symptoms measured using the Beck Depression Inventory between the active and placebo groups^(^^63^^)^.

Another interpretation of the present results is that vitamin D supplementation might only work in participants with low serum 25-hydroxyvitam D or high depressive symptoms. Several of the previous supplementation trials investigated the benefit of supplementation in samples with low baseline serum 25-hydroxyvitamin D. By contrast, ours was a relatively healthy sample, and even though participants were, on average, at the higher end of anxiety, it was still a non-clinical sample. However, there was variability in the present sample, with some women starting off with low baseline vitamin D and higher depressive symptoms. When we tested whether these baseline characteristics affected the intervention effects, albeit in a minimised sample, we found no evidence that low baseline serum 25-hydroxyvitamin D concentration or high starting depression symptom scores were a factor. It is possible that these tests were underpowered, and that more targeted recruitment based on low vitamin D concentration and/or high depressive symptoms would have allowed further investigation of this hypothesis.

Furthermore, the duration of the supplementation trial could influence the outcomes. Participants in the present study were supplemented over 6 months from late summer/autumn to early spring. This is the time when serum 25-hydroxyvitamin concentration declines due to the lesser efficiency of endogenous vitamin D manufacture as people tend to spend less time outdoors, wrap up more in the cold, and the UV rays are not at an optimal angle. Even though the study was conducted during winter to prevent worsening of mood, it is possible that a longer supplementation period is required for serum 25-hydroxyvitamin D to show any effect on the mood measures. Lansdowne & Provost observed a difference in moods between the supplemented and placebo groups after a mere 5 d supplementation protocol^(^^34^^)^. In contrast, Sanders *et al.* found no benefit of vitamin D supplementation on mental health with a single yearly high dose administered to a non-clinical sample in autumn for 3 to 5 years^(^^35^^)^. Due to the length of the trial (3- to 5-year supplementation period) with a yearly dose, participants may have been vitamin D replete, and the upcoming dose did not make a difference. However, the authors found no association between 25-hydroxyvitamin D and mental health outcomes at any point in the study.

Some of the other outcomes of sun exposure not achieved with vitamin D supplementation include an increase in the production of NO in response to exposure to frequencies in the UVA spectrum. NO is in turn associated with an increased cerebral blood flow, improving cognitive functioning^(^^64^^)^. As a result, the mechanism of association between serum 25-hydroxyvitamin D levels and psychological states like depression and mood are not necessarily straightforward. In a free-living population, it would be challenging to control for the exposure to particular light frequencies. Although time spent outdoors was accounted for in the present study, the exact time and specifics of being outdoors were unmeasured and unknown.

### Strengths and limitations

A major strength of the study was the randomised double-blind placebo-controlled design which administered a sufficient vitamin D dose that was effective in preventing a seasonal decline in vitamin D status and resulted in 25-hydroxyvitamin D levels that were significantly higher than in the placebo group. The length of the trial may be a potential limitation of the study in addition to the dosing regimen employed, and the sample including only women. The main limitation of the present study is that the majority of the sample consisted of European women with no vitamin D deficiency or high depressive symptoms. This limits the generalisability of findings beyond this population. We also have limited information about participant adherence, as only about two-thirds of participants returned their supplement containers; however, the pre-winter concentration of 25-hydroxyvitamin D was maintained in the treatment group, suggesting adequate adherence.

### Conclusion

Biological evidence suggests that vitamin D plays an important role in the brain; however, findings from the present randomised double-blind placebo-controlled trial, which included a wide range of mood outcomes, did not demonstrate an effect of supplementation on any mood outcomes assessed. These results suggest that vitamin D supplementation to prevent depressive symptoms and anxiety, or improve flourishing or mood, for healthy adult women over winter may not be warranted. Future trials should investigate other groups of individuals, for example men, those with clinical depression, or low vitamin status.
